# Preparation and Characterization of Silver-Iron Bimetallic Nanoparticles on Activated Carbon Using Plasma in Liquid Process

**DOI:** 10.3390/nano11123385

**Published:** 2021-12-14

**Authors:** Heon Lee, Jaegu Park, Young-Kwon Park, Byung-Joo Kim, Kay-Hyeok An, Sang-Chai Kim, Sang-Chul Jung

**Affiliations:** 1 Department of Environmental Engineering, Sunchon National University, Suncheon 57922, Korea; honylee@hanmail.net (H.L.); worn0623@gmail.com (J.P.); 2 School of Environmental Engineering, University of Seoul, Seoul 02504, Korea; catalica@uos.ac; 3 Department of Carbon & Nanomaterials Engineering, Jeonju University, Jeonju 55069, Korea; kimbj2015@gmail.com (B.-J.K.); khandragon@jj.ac.kr (K.-H.A.); 4 Department of Environmental Education, Mokpo National University, Muan-gun 58554, Korea; gikim@mokpo.ac.kr

**Keywords:** plasma in liquid process, Ag-Fe/AC composite, bimetallic nanoparticles, precursor

## Abstract

The mono and bi-metallic nanoparticles have conspicuous properties and are widely used in the environment, energy, and medical fields. In this study, bimetallic nanoparticles composed of silver and iron were precipitated on the surface of activated carbon in a single process using plasma in liquid process (PLP). Silver-iron ions and various radicals were actively generated in the aqueous reactant solution by the PLP. Although metals were precipitated on AC depending on the number of precursors added to the aqueous reactant solution, the standard reduction potential of silver ions was higher than that of iron ions, so silver precipitated on AC. The silver precipitate on AC was a mixture of metallic silver and silver oxide, and iron was present as Fe_3_O_4_. Spherical nanoparticles, 100–120 nm in size, were observed on the surface of the Ag-Fe/AC composite. The composition of the bimetallic nanoparticles could be controlled by considering the ionization tendency and standard reduction potential of metal ions and controlling the concentration of the precursors. The PLP presented in this study can be applied to the preparing method of bimetallic nanoparticle/carbon materials and can be expected to be used in the prepare of energy and environmental materials such as MFC and absorption materials for removing pollutants.

## 1. Introduction

Advances in nanoscience are having a drastic impact in many fields. In particular, nanoparticles are applied to catalysts and batteries for use in the environment and energy fields [1,2]. Silver nanoparticles are attracting attention as new high-tech materials with high added value, and interest is focused on antibacterial substances, antistatic agents, cryogenic superconductors, and biosensors [3,4]. Although iron nanoparticles can be prepared inexpensively, they are used widely in environmental purification and recently as an additive for energy materials [5,6]. Recently, silver–iron bimetallic nanoparticles have attracted attention because they can be applied to microbial fuel cells (MFCs) using the oxygen reduction reaction (ORR) [7,8].

Carbon materials, such as activated carbon, graphite, carbon black, graphene, and carbon nanotubes, are replacing platinum/carbon catalysts because of their relatively low cost, specific structure, and redox activity [9,10]. In addition, many attempts have been made to load a low-cost metal with excellent catalytic efficiency into a carbon material, among which the carbon catalyst in which iron is precipitated showed excellent electron-donating ability as an MFC cathode [11,12]. On the other hand, silver nanoparticles deposited on carbon materials are being used as ORR electrocatalysts with the ability to inhibit bacteria [13,14].

Recently, the plasma in liquid process (PLP) has attracted attention as a useful method of depositing various metals on carbon materials [15,16]. Various metal/carbon composites are prepared by the PLP because it is easy to perform and can precipitate metal on carbon material easily in a single step [17,18].

In this research, silver and iron were precipitated simultaneously on activated carbon using PLP in a single step. The physicochemical properties of the Ag-Fe/AC composite materials synthesized by PLP were investigated through various instrumental analyses. Furthermore, the composition of bimetallic nanoparticles could be controlled.

## 2. Materials and Methods

### 2.1. Materials and Chemicals

Activated carbon (YP-50F, Kuraray chemical Co. Ltd., Osaka, Japan) was used as the composite substrate. Iron (III) nitrate nonahydrate (Fe(NO_3_)_3_·9H_2_O, Sigma-Aldrich, St. Louis, MO, USA) and silver nitrate (AgNO_3_, Sigma-Aldrich, St. Louis, MO, USA) were used as the silver and iron precursors for the bimetallic nanoparticles (BNPs). Deionized water with an electrical conductivity of less than 2 μS/cm from Daejung Chemical & Metal Co. Ltd. was used to prepare the aqueous reactant solution required for the PLP reaction. 

### 2.2. Device

**Figure 1:**
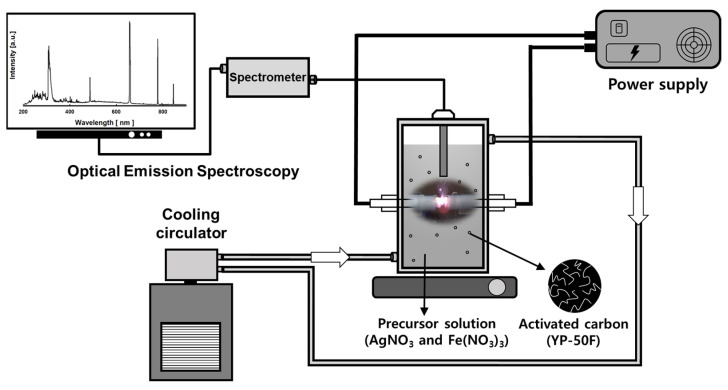
Diagram of the PLP reaction device for preparing Ag/Fe bimetallic nanoparticles supported on activated carbon composites.

Figure 1 presents a schematic diagram of a PLP device for preparing silver-iron bimetallic nanoparticles precipitated on activated carbon composites (SIACCs); details of each part are described in detail elsewhere [15,17]. The two-channel PLP reactor was made of Pyrex, the aqueous reactant solution was filled inside, and the cooling water supplied from a chiller was circulated in the outside channel. Cooling water (ethylene glycol 40%) was circulated to prevent the increase in temperature of the aqueous reactant solution caused by the heat of the plasma generated by the tungsten electrodes and maintain a constant reaction temperature. A sensor of optical emission spectroscopy (OES, AvaSpec-3500, Avantes, Apeldoorn, The Netherlands) was installed over the contacts of the tungsten electrodes to collect plasma field information. The distance between the plasma field and the OES sensor was maintained at 3 mm. 

Cylindrical rod type tungsten electrodes (ϕ 2mm, L 150 mm, 99.95%, Wolfram industrie, Traunstein, Germany) used to generate the plasma were installed facing each other at a 1 mm interval in the center of the PLP reactor. The outside of the tungsten electrode was insulated using a ceramic insulator and PTFE tube. The power supply (NTI-1000w, Nanotechnology Inc., Daejeon, Korea) was a high-frequency bipolar pulse type in which the frequency, applied voltage, and pulse width could be altered. The BNPs were prepared under the following operating conditions: frequency of 30 kHz, applied voltage of 250 V, and pulse width of 5 μs.

### 2.3. Preparation of SIACCs

**Figure 2:**
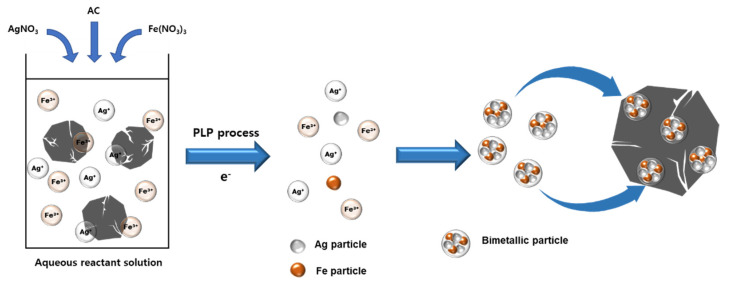
Schematic illustration of bimetallic nanoparticle formation and SIACC preparing using PLP method.

Figure 2 is a schematic illustration showing the bimetallic nanoparticle formation and SIACC preparing process using PLP. Silver ions and iron ions present in the reactant solution due to the dissociation of the precursor undergo particle formation and growth by PLP and are then precipitated to the activated carbon surface as bimetallic nanoparticles. The SIACCs were prepared using the PLP is as follows. Ag and Fe precursors were added to 250 mL of deionized water at a certain concentration ratio and dissolved by stirring. After adding 0.5 g of activated carbon (AC) as a substrate to the aqueous reactant solution in which the precursor was dissolved and stirred for 10 min, the prepared aqueous reactant solution was placed in the reactor shown schematically Figure 1. PLP was performed for one hour by receiving power from a power supply. After the reaction, the precipitate was separated from the reactant by centrifugation. The separated sediment was centrifuged and washed three times, and a final product (SIACCs) was obtained by filtration, and moisture was removed by vacuum drying at 353 K for 24 h.

### 2.4. Characterization of SIACCs 

The chemical composition of the SIACCs prepared using PLP was analyzed using a Field Emission Scanning Electron Microscope (FESEM, JSM-7100F, JEOL, Tokyo, Japan) equipped with energy dispersive X-ray spectroscopy (EDS, Noran Z-MAX 350, Tokyo, Japan), and the morphology and constituent elements of silver-iron bimetallic nanoparticles (BNs) precipitated on the AC surface were observed using a field emission transmission electron microscope (FETEM, JEM-2100, JEOL, Tokyo, Japan). The chemical state and bond formation of SIACCs were measured by X-photoelectron spectroscopy (XPS, Multilab 2000 system, Thermo Fisher Scientific, Waltham, MA, USA). The diffraction data of SIACCs prepared by PLP were measured with a high-resolution X-ray diffractometer (HR-XRD, Max Ultima III, Rigaku, Austin, TX, USA). 

## 3. Results and Discussion

### 3.1. Characteristics of Aqueous Reactant Solution

**Figure 3:**
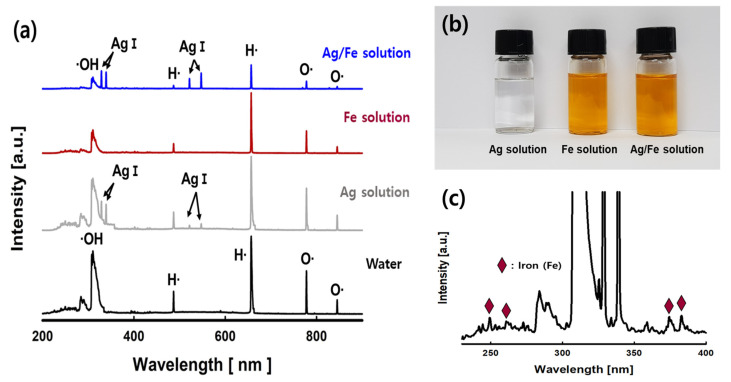
OES spectra of the aqueous reactant solution with metal precursors (**a**), aqueous reactant solutions after PLP reaction (**b**), and OES spectra in the range of 200–400 nm of Ag/Fe aqueous reactant solution (**c**).

The optical spectra generated in the aqueous reactant solution were observed to confirm the chemical species generated during the PLP reaction. Figure 3a shows the OES spectra in the range of 200 to 900 nm for DI water, silver, iron, and silver-iron solutions. The concentrations of silver and iron precursors used were 1 mM and 10 mM, respectively. Five strong peaks were observed in the OES spectra of water shown at the bottom: hydroxyl radicals (309 nm), hydrogen radicals (486 nm and 656 nm), and oxygen radicals (777 nm and 844 nm) [19]. The radical peaks (hydroxyl, hydrogen, and oxygen) generated in the reactant aqueous solution in which the silver precursor (AgNO_3_, 1 mM) is dissolved show a slightly decreased intensity than those generated in water. Meanwhile, new peaks were observed at 328.0 nm, 338.2 nm, 520.9 nm, and 546.5 nm, which were assigned to Ag I (Ground state: 1*s*^2^2*s*^2^2*p*^6^3*s*^2^3*p*^6^3*d*^10^4*s*^2^4*p*^6^4*d*^10^5*s*^2^S_1/2_) generated in the aqueous reactant solution by the PLP reaction [20]. In the case of the OES spectra of the reactant aqueous solution in which the iron precursor (Fe(NO_3_)_3_, 10 mM) was dissolved, the intensity of the radical peaks observed in the water rapidly decreased. The OES spectra of the reactant aqueous solution are measured by the light emitted during plasma reaction, and the intensity of the generated peaks is affected by the transparency of the reactant solution. Figure 3b presents a photograph of the color change of the solution after performing the PLP reaction for one minute on an aqueous reactant solution in which the metal precursor was dissolved. The Ag solution remained colorless and transparent as before the PLP reaction, but the aqueous reactant solution (Fe solution and Ag/Fe solution) containing the iron precursor changed to brown. This was attributed to the formation of iron oxide (Fe_3_O_4_) nanoparticles by the PLP reaction. In a previous study, iron oxide nanoparticles were not produced when an aqueous reactant solution was prepared using ethanol, but some iron oxide nanoparticles and iron nanoparticles were produced when an aqueous reactant solution was prepared using water [21]. The decrease in intensity of radical peaks in the OES results of the iron precursor reactant aqueous solution is presumed to be due to the transparency caused by the color change of the reactant solution. Even in the case of OES of a reactant aqueous solution containing silver and iron precursors, the intensity of radical peaks decreased sharply, and peaks caused by Ag I were observed. 

It was confirmed that the characteristics of Ag solution and Fe solution were simultaneously expressed by the plasma generated in the reactant aqueous solution. Figure 3c shows an enlarged portion of the OES spectra of Ag-Fe aqueous reactant solution, and peaks by Fe I (249.0 nm, 344.0 nm, 374.9 nm, and 382.0 nm) and Fe II (260.7 nm) were observed [20,22]. Silver ions (Ag^+^) and iron ions (Fe^3+^) present in the aqueous reactant solution generate various active peaks in the PLP reaction.

### 3.2. Properties of Silver-Iron Bimetallic Nanoparticles

**Figure 4:**
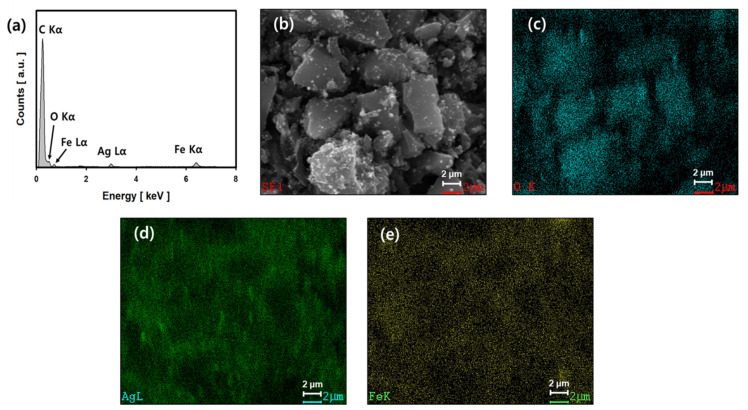
EDS spectrum (**a**), FE-SEM real image (**b**), oxygen (**c**), silver (**d**), and iron (**e**) elemental mapping result of SIACC prepared using the PLP method.

Figure 4a shows the EDS spectrum attached to the FE-SEM for the SIACC composite prepared by mixing the precursor concentration ratio of Ag and Fe at 1:50. The strong peak at 0.25 keV is due to carbon (C Kα), which is a major constituent element of activated carbon. The weak peak at 0.53 keV is due to oxygen (O Kα). The peak observed at 2.98 keV is silver (Ag Lα), and the peaks at 0.70 keV and 6.39 keV are peaks due to Lα and Kα of iron (Fe). From these results, silver and iron precipitated in the composite by PLP. Figure 4b shows the real image of FE-SEM, and Figure 4c–e is an elemental mapping image showing the distribution of oxygen, silver, and iron elements in the region of Figure 4b. In Figure 4c, it can be seen that oxygen in SIACC has the same distribution as that of AC. From Figure 4d,e, it can be seen that the silver and iron elements are uniformly dispersed in the SIACC composite and show a relatively similar shape.

Table 1 shows the initial precursor concentration and chemical composition of SIACCs using pristine AC (YP-50F) prepared by PLP. The Fe(NO_3_)_3_·9H_2_O concentration in the aqueous reactant solution was kept constant at 10 mM, and the silver nitrate (AgNO_3_) concentration was changed to 0.1–1.0 mM. YP-50F is mostly composed of carbon with 3.6 wt.% oxygen. The chemical composition of SIACCs prepared by PLP was changed by the precursor concentration of Ag and Fe. In the case of SIACC-10, the ratio of silver and iron precursors was 1:10. On the other hand, the silver and iron content in the resulting composite was 3.0 wt.% and 0.8 wt.%, respectively, indicating a higher silver content than iron. As shown in Figure 2, active species and electrons are generated from the plasma field generated by PLP. Silver ions and iron ions that existed in an ion state in the reactant aqueous solution are reduced by electrons and changed into metallic particles. Bimetallic particles composed of silver and iron elements that are continuously reduced by electrons generated in the PLP reaction are precipitated on the AC surface through the generation and growth. The reduction rate of metal ions is affected by the standard reduction potential (SRP). The SRP of Ag^+^ is +0.799 V, which is higher than Fe^2+^ (−0.04 V). Therefore, silver reduced faster than iron and more silver precipitated on the AC [23,24,25]. The iron nitrate used in this study can cause etching on silver metal. In this experiment, an aqueous reactant solution was prepared by mixing silver nitrate (0.1–1 mM) and iron nitrate (10 mM) at a low concentration, and bimetallic particles were prepared by simultaneous reduction of two metal ions by plasma reaction. As can be seen from the results in Table 1, the etching effect by iron nitrate hardly occurred, and it was found that the chemical composition was determined by the SRP of the metal ions. In addition, the oxygen content of the SIACCs prepared by the PLP reaction was higher than that on bare AC, which was attributed to oxides included in the BNPs generated on the AC surface.

**Figure 5:**
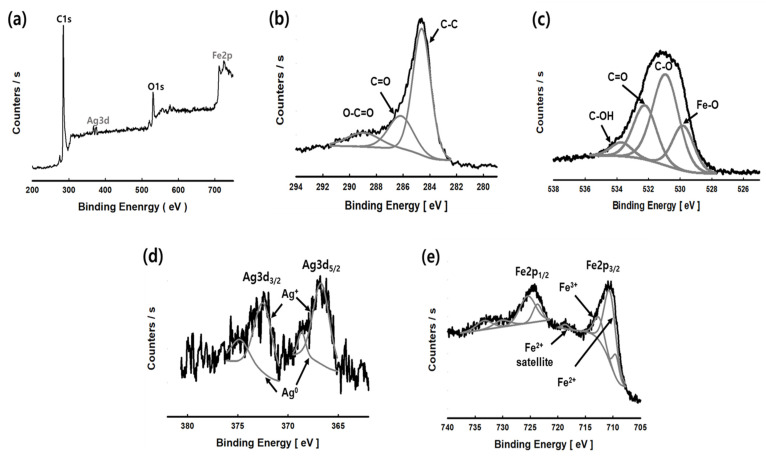
Survey spectrum (**a**), C1s region (**b**), O1s region (**c**), Ag3d region (**d**), and high-resolution XPS spectra of Fe2p region (**e**) of SIACC-02 prepared by the PLP process.

Figure 5 shows the XPS spectrum measured to confirm the chemical state and bond formation of SIACC-02. The survey spectrum in Figure 5a shows that C1s and O1s peaks are generated at 284 eV and 530 eV, respectively, which are constituent elements of AC, and Ag3d (BE 367 eV and 372 eV) and Fe2p (BE 711 eV and 724 eV) showed that Ag and Fe had precipitated on the AC surface through the PLP reaction. In the spectrum of C1s region in Figure 5b, the strong peak of BE 284.6 eV is due to sp^2^-hybridized graphitic carbon (C-C group), and the peaks at BE 286.2 eV and BE 288.9 eV are hydroxyl/epoxy group (CO) and it is due to the carbon of the carboxyl group (O-C=O) [26,27,28]. All three peaks are due to carbon of AC, and no new peaks were observed [29]. Figure 5c is the spectrum for the O1s region. BE 530.5 eV (C-O group), 532.2 eV (C=O group), and 533.8 eV (C-OH group) were peaks produced by oxygen combined with AC and were identical to the O1s region results of AC (YP-50F) [30]. In addition, the weak peak observed at BE 529.8 eV is due to O^2−^ of the Fe-O group, indicating oxygen bound to iron in BNPs [26]. In the Ag3d region of Figure 5d, two peaks were observed in Ag3d_3/2_ and Ag3d_5/2_, respectively. The peaks at BE 367.7 eV and 372.5 eV were due to Ag^+^ of Ag_2_O, and the peaks at BE 368.2 eV and 374.7 eV were due to metallic silver (Ag^0^) [31,32]. Therefore, the Ag nanoparticles on the AC surface by PLP are a mixture of metallic silver and silver oxide. Figure 5e shows the results for the Fe2p region. The peaks due to Fe 2p_3/2_ and Fe2p_1/2_ were observed at BE 724.3 eV and 710.7 eV, respectively. The interval between the two peaks was 13.6 eV. Hence, the peak was assigned to Fe_3_O_4_ [33]. The BE 710.8 eV and BE 712.7 eV of Fe2p_3/2_ were peaks generated by Fe^2+^ and Fe^3+^, The peak shown at BE 718.7 eV is a shake satellite peak for Fe ion of divalent sate [34,35,36]. Therefore, the BNPs on the AC surface by the PLP reaction exist in the form of a mixture of Ag, Ag_2_O, and Fe_3_O_4_. The atomic percentages (At.%) of Ag and Fe elements of SIACC-02 measured through XPS analysis were 0.24 At.% (1.92 wt.%) and 0.95 At.% (4.12 wt.%), respectively, which were higher than the data obtained by EDS analysis (Table 1). In general, XPS is applied for surface analysis rather than bulk material analysis because of its low sampling depth (1–10 nm). The increase in Ag and Fe values in XPS analysis compared to EDS analysis means that there are many Ag and Fe elements on the surface of AC, and it is because BNPs generated by PLP were mainly precipitated on the AC surface.

**Figure 6:**
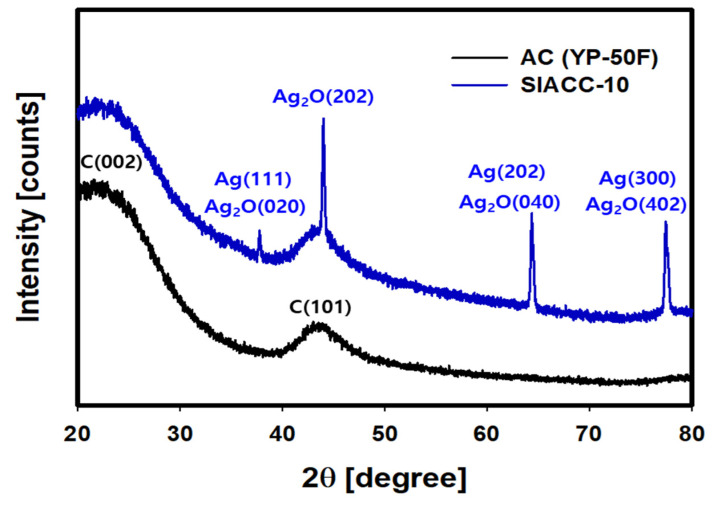
XRD pattern of SIACC-10 prepared by PLP reaction and Bare AC (YP-50F).

Figure 6 shows the XRD patterns of AC (YP-50F) and SIACC-10. The XRD pattern of AC (YP-50F) showed broad peaks at 22° and 43° 2θ. The broad XRD peak centered at 22° 2θ was assigned to the 002 plane of parallel graphite flakes, and the peak at 43° 2θ was due to the 101 planes of the honeycomb structure. The broad XRD peak indicated that it was amorphous [37,38]. In the XRD pattern of SIACC-10 prepared by PLP, sharp peaks were also observed at 38.1, 44.2, 64.4, and 77.4° 2θ, which were assigned to Ag (111 plane) and Ag_2_O (020 plane), Ag_2_O (202 plane) [39,40], Ag (202 plane) and Ag_2_O (040 plane), and Ag (300 plane) and Ag_2_O (402 plane), respectively [39]. Hence, Ag precipitated on the AC by the PLP reaction as a mixture of Ag and Ag_2_O. On the other hand, in the XRD pattern of SIACC-10, no peak for iron was observed, which is because the chemical composition of the iron was below the detection limit XRD.

### 3.3. Controlling the Chemical Composition of Bimetallic Nanoparticle

**Figure 7:**
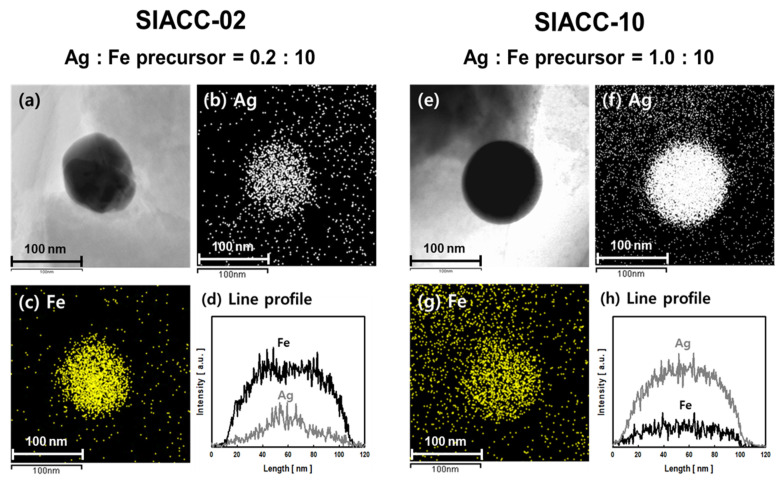
Real images (**a**,**e**), Ag element mapping images (**b**,**f**), Fe element mapping images (**c**,**g**), and line scanning profiles (**d**,**h**) of bimetallic nanoparticles (SIACC-02 (**a**–**d**) and SIACC-10 (**e**–**h**)) observed by FE-TEM.

Figure 7 shows the morphology and components of the nanoparticles precipitated on the AC surface by PLP using FE-TEM. Figure 7a,e includes real images of nanoparticles of SIACC-02 and SIACC-10. The precipitated nanoparticles were spherical, approximately 100–120 nm in size. Figure 7b,c shows the elemental maps of Ag and Fe of SIACC-02. More Fe was present than Ag. Hence, more Fe precipitated than Ag, which is consistent with the results in Table 1. Figure 7d shows the line scanning profile for the BNP of Figure 7a. The intensity by Fe was higher than that of Ag, showing a high concentration in the whole part of the particle. Figure 7e–h shows a real image, elemental mapping, and line scanning profile of the nanoparticles precipitated from the SIACC-10 aqueous reactant solution. Figure 7f,g includes the Ag and Fe element mapping results. The content of Ag was higher than that of the Fe, possibly due to the increase in Ag precursor concentration in the aqueous reactant solution. The concentration of the Fe precursor in the aqueous reactant solution of SIACC-02 and SIACC-10 was the same (10 mM), and the initial concentration of Ag precursor was increased slightly from 0.2 mM to 1 mM. Hence, the reduction of silver ions occurred preferentially in the PLP reaction. Figure 7h shows the line scanning profile of BNP precipitated in the SIACC-10 aqueous reactant solution. Compared to the Fe element, the intensity of Ag was larger in the entire part of the particle. These results suggest that the metal precursor ratio in the reaction solution is an important factor in the chemical composition of BNPs in the generation of BNPs through the PLP reaction. In conclusion, the composition of bimetallic nanoparticles can be controlled by considering the ionization tendency and standard reduction potential of the metal ions and adjusting the precursor concentration.

## 4. Conclusions

Silver and iron were precipitated on AC powder in a single step using PLP. OES confirmed silver and iron ions and various radicals in the reaction aqueous solution. EDS quantified the content of elements present in SIACC. Although metals were precipitated on AC depending on the amounts of precursors added to the aqueous reactant solution, the standard reduction potential of silver was higher than that of iron, so relatively more silver precipitated on AC powder. XPS confirmed that silver precipitated on AC powder as a mixture of metallic silver and silver oxide and iron as Fe_3_O_4_. FE-TEM showed that the BNP on the surface of the SIACC were spherical, 100–120 nm in size. Elemental mapping and line scanning profile revealed bimetallic particles containing silver and iron, and the chemical composition could be controlled by changing the concentration of the initial precursors. It can be said that this study suggested a method for preparing a carbon composite incorporating BNPs and controlling the composition of BNPs. These research results can be applied to MFC and materials for removing environmental pollutants, and we plan to conduct research on performance improvement in future studies.

## Figures and Tables

**Table 1:** Chemical composition of SIACCs using pristine AC prepared by PLP.

Sample	Initial Conc. (mM)		Carbon	Oxygen	Silver	Iron
	AgNO_3_	Fe(NO_3_)_3_	wt.%	wt.%	wt.%	wt.%
AC(YP-50F)	0	10	96.4 ± 0.3	3.6 ± 0.3	0.0 ±0.0	0.0 ± 0.0
SIACC-01	0.1	10	93.2 ± 0.2	4.6 ± 0.2	0.3 ± 0.1	2.0 ± 0.2
SIACC-02	0.2	10	93.1 ± 0.4	4.4 ± 0.2	0.7 ± 0.1	1.7 ± 0.1
SIACC-10	1.0	10	91.9 ± 0.4	4.3 ± 0.3	3.0 ± 0.2	0.8 ± 0.1

## Data Availability

The data presented in this study are available on request from the corresponding author.
